# Commentary: Prestimulus Theta Oscillations and Connectivity Modulate Pain Perception

**DOI:** 10.3389/fnhum.2016.00535

**Published:** 2016-10-26

**Authors:** Giuseppe Spinelli, Valentina Nicolardi

**Affiliations:** ^1^Department of Psychology, Sapienza University of RomeRome, Italy; ^2^IRCCS Santa Lucia FoundationRome, Italy

**Keywords:** feedback processing, gamma oscillations, pain perception, salience processing, theta oscillations

Pain experience includes the fine-grain integration of both attentive and automatic (bottom-up; Legrain et al., 2012), as well as affective and intentional (top-down; Buschman and Miller, 2007) processes. While the neural underpinnings of post-stimulus pain processing have been deeply explored (Hauck et al., 2008), the oscillatory brain activity preceding pain processing is less far investigated.

In a recent issue of Journal of Neuroscience, Taesler and Rose (2016) addresses this topic by recording EEG from healthy participants who were forced to judge as “painful” or “non-painful” series of electrical stimulations delivered on their own left-hand. Painful stimulation was elegantly kept constant at the painful-threshold of each participant, and a subtractive approach (“painful” minus “non-painful” trials) was adopted to study the EEG signatures underlying pain processing. Results revealed enhanced pre-stimulus theta and gamma activity. Specifically, pre-stimulus theta power was both increased and decreased over fronto-temporal ipsilateral and contralateral electrodes, respectively. Instead, pre-stimulus gamma power was enhanced over frontal and temporal electrodes. Tellingly, while the ipsilateral reduction of theta-band power paralleled a decreased theta-band connectivity over frontal-lateral electrodes, the contralateral increase resulted in an enhanced connectivity of a bilateral fronto-temporal/centro-parietal network. Also, the connectivity-pattern of the gamma increased between Fz and a parieto-temporal cluster around P6. Interestingly, source-analysis revealed likely generators in the bilateral insular cortex and posterior cingulate cortex for the theta, and contralateral insular cortex for the gamma band.

While this paper provides interesting and novel findings, we believe that these results deserve to be further discussed and interpreted in view of (i) the state-of-art literature of pain, and (ii) an integrated neurocognitive model of that takes into account both pain and negative-feedback processing.

First, the core of discussion of the authors aims at interpreting pre-stimulus theta oscillations as the vehicle by which both contra- and ipsi-lateral insular cortices communicate to process painful stimulation. This relevant result is compared to two previous studies (Sarnthein et al., 2006; Walton et al., 2010) on chronic-pain patients, in which, however, only post-stimulus theta oscillations were related to pain processing. Thus, while Taesler and Rose provided relevant additions to the literature on pain processing, a direct comparison with these latter studies is still difficult to be assessed given the different neurocognitive mechanisms underlying the processing of pain in individuals with chronic sensitization respect to healthies (Bushnell et al., 2013).

Moreover, the pre-stimulus theta-band power increase can be viewed from a different perspective when considering that the paradigm of Taesler and Rose, by delivering in each trial a constant painful stimulation after a fixation-cross, induces a strong “stimulus–stimulus” association. This trial-by-trial “stimulus–stimulus” reinforcement, can induce an expectancy of pain that might lead the neurocognitive system of pain processing to be active before the onset of the actual painful stimulation. Such an affective and anticipatory activation is in keeping with previous evidences (Palermo et al., 2015; Sawamoto et al., 2000) showing that expectation of pain amplifies brain responses to the somatosensory stimulation and activates mainly the anterior cingulate cortex and insular cortices.

Consequently, given that both insular cortices and various regions of the middle-frontal cortex (Ridderinkhof et al., 2004), are linked to pre- and post-conflict monitoring (Yeung et al., 2004), feedback-monitoring and reinforcement-learning (Holroyd and Coles, 2002) via increasing theta band oscillations (Cohen, 2014), we argue that the pain processing system can share the same temporal dynamics as the performance (and conflict) monitoring system (Shackman et al., 2011). In fact, it is worth noting how in Taesler and Rose the displacement of theta oscillations from pre- to post-stimulus, evolves from insular cortices to fronto-central and dorso-lateral ones. In this view, we suggest that the temporal dynamics of processing painful stimulation can develop from an early affective, orienting/salience response to unpleasant/unfavorable outcomes—processed by right and left insular cortices prior to painful stimulation—to a late processing of the unfavorable/painful outcome itself—processed by frontal regions after painful stimulation. Accordingly, the theta-related connectivity pattern showed by Taesler and Rose, seems to resemble the pattern of activation usually found when the so-called “saliency network”—mostly governed by the anterior insular cortex—attempt to elicit a (re)orienting response before negative outcomes. In fact, it is arguably known how the activation of the anterior insular activity, by signaling an increased likelihood to receive unfavorable outcomes, aims at rapidly calling for additional cognitive control or behavioral adjustment (Klein et al., 2007). Thus, considering that in Taesler and Rose, participants were always stimulated at their own painful threshold, the activity in the insular cortices could have governed, via theta increase, two different processes, namely: (i) the integration of personally/motivationally information linked to painful stimulation and (ii) the preparatory allocation of cognitive and physical resources of salience processing. While the latter is in keeping with previous evidence (Sridharan et al., 2008), the former can be addressed to the notion that pain-related cortical responses reflect the processing of saliency of painful stimulations that is related to a salience-specific multimodal neural activity rather than pain-specific unimodal neural response (Valentini et al., 2011). However, studies investigating this latter issue in the pre-stimulus interval are still missing, and further effort is needed to explore the link between these two cognitive processes and the underlying electro-cortical signatures of pain processing.

Contrarily, the later stage of pain detection and the post-stimulus theta synchronization over middle-frontal and parieto-occipital brain regions, has been less discussed by the authors. It is worth mentioning that the pre-stimulus theta activation over the bi-lateral insular cortices shifts over fronto-central scalp location during the post-stimulus processing. Interestingly, such an increase over mid-frontal electrodes might be linked to the neurophysiological coding of: (i) individual psychological traits during processing of painful stimuli (Schulz et al., 2012), and (ii) negative outcome monitoring related to a recruitment of top-down cognitive processes to ultimately adapt future behavior. This latter hypothesis needs to be tested as it could represent a valid integration of the saliency-processing theory (Iannetti and Mouraux, 2010) and the performance-monitoring model. In this view, painful stimulation might be processed by the brain in the same way as negative feedback/outcome (Shackman et al., 2011), and its detection and encoding may be reflected in a coupling between post-stimulus theta-gamma oscillations, where theta might be considered an high-level/ cognitive cortical marker of mismatch detection, and the gamma a low-level/perceptual neural signature of the saliency and the intensity of pain processing, respectively.

Concluding, Taesler and Rose provided optimal evidences to deepen the functional significance of theta oscillations evoked before and after painful stimulation. Based on our arguments we also believe that future investigations should focus on an integrative neuro-cognitive model of pain perception that should take into account also negative/unpleasant outcome monitoring, performance monitoring models, and their underlying electro-cortical signatures.

## Author contributions

GS and VN equally contributed to read, comment, and write the commentary.

### Conflict of interest statement

The authors declare that the research was conducted in the absence of any commercial or financial relationships that could be construed as a potential conflict of interest.
